# Analyzing the Potential of Drill Bits 3D Printed Using the Direct Metal Laser Melting (DMLM) Technology to Drill Holes in Polyamide 6 (PA6)

**DOI:** 10.3390/ma16083035

**Published:** 2023-04-12

**Authors:** Lukasz Nowakowski, Michal Skrzyniarz, Slawomir Blasiak, Jaroslaw Rolek, Dimka Vasileva, Tanya Avramova

**Affiliations:** 1 Department of Machine Design and Machining, Kielce University of Technology, 25-314 Kielce, Poland; lukasn@tu.kielce.pl (L.N.); mskrzyniarz@tu.kielce.pl (M.S.); 2 Department of Industrial Electrical Engineering and Automatic Control, Kielce University of Technology, 25-314 Kielce, Poland; jrolek@tu.kielce.pl; 3 Department of Manufacturing Engineering and Machine Tools, Technical University of Varna, 9010 Varna, Bulgaria; d.vasileva@tu-varna.bg (D.V.); tanya_avramova@tu-varna.bg (T.A.)

**Keywords:** additive technology, drilling, cutting force and torque, direct metal laser melting

## Abstract

Drilling with standard twist drill bits is the most common method to create cylindrical holes. With the constant development of additive manufacturing technologies and easier access to additive manufacturing equipment, it is now possible to design and fabricate solid tools suitable for various machining applications. Specially designed 3D printed drill bits seem more convenient for standard and nonstandard drilling operations than conventionally made tools. The study described in this article aimed to analyze the performance of a solid twist drill bit made from steel 1.2709 using direct metal laser melting (DMLM), which was compared with that of a drill bit manufactured conventionally. The experiments involved assessing the dimensional and geometric accuracy of the holes made by the two types of drill bits and comparing the forces and torques occurring during the drilling of holes in cast polyamide 6 (PA6).

## 1. Introduction

Additive manufacturing (AM), also known as rapid prototyping (RP) or 3D printing, is a group of modern manufacturing processes able to create geometrically complex objects from 3D CAD data with little or no need for finishing operations. AM is increasingly common in the automotive, [1,2], aviation [3], biomedicine, medicine [4], food processing, maintenance and repair, education, and entertainment sectors. Like any other manufacturing process, AM has some shortcomings. The limitations relate to the types of materials, production capacity, surface quality, and post-processing operations [5]. The major advantage of AM is the possibility to create a ready-to-use part with final dimensions in one step. This, however, makes the process quite expensive.

Over the years, many studies have focused on the design and manufacture of finished elements in just one process [6]. An important variety of RP is rapid tooling (RT), which allows us to fabricate tools quickly. As described in [7], tools created through direct metal laser sintering can be improved by filling pores with high temperature epoxy resin to ensure higher compressive strength. Additionally, electroless nickel plating can be used to increase the material hardness and resistance to wear and abrasion; this, however, has no effect on the dimensional and geometric accuracy of the tool.

Despite rapid technological development, drilling is still the most common and best-known method to make cylindrical holes. Drilling with additively manufactured tools is, however, a new solution, which requires researchers to investigate it thoroughly.

Generally, the drilling process is expected to ensure high dimensional and geometric accuracy and low surface roughness of the holes. This, however, may not be easy to achieve with some materials. Problems have been observed, for instance, for Ti–6Al–4V, which is difficult to machine due to low thermal conductivity and high specific strength. Drilling holes in this alloy using tungsten carbide (WC) or high-speed steel (HSS) drill bits is difficult because the higher the cutting speed, the higher the friction between the tool and the workpiece and, consequently, the higher the temperature of the cutting tool, i.e., the drill bit. Higher feed rates, on the other hand, are responsible for higher tool wear, which results in higher surface roughness (arithmetic average roughness (Ra)). The process parameters also affect the hole diameter, which increases with increasing feed rate [8,9,10]. When, however, there is an increase in the cutting speed, the mean diameter error is smaller [11]. It is also reported that the hole diameter at the inlet is greater than in the central part [12]. Another interesting finding in this area is the influence of the cutting speed and the cutting length on the variability of the cutting forces and the quality of the holes. The reason for that is most probably the wear of the tool and the changes in the tool geometry. Changes in the geometry of the cutting edges may lead to deformations of the hole surfaces [13]. At larger helix angles and higher cutting speeds, the tool wear increases due to higher friction and larger heat flux, both of which are a result of adhesion and diffusion. Vibration generated in the workpiece–tool system is another important factor affecting the hole drilling process, it increases with the increasing helix angle and cutting speed [14]. Vibration is responsible for higher surface roughness (Ra) [15].

The use of a cutting fluid has a considerable effect on the hole roundness and the forces taking part in the cutting process [16,17].

The chip formation process may look different for different materials. For the majority of metal alloys and plastics, formation of discontinuous chips is the most favorable. For some materials, higher feed rates in drilling result in shorter chip lengths, while higher cutting speeds do not affect the hole drilling process [18].

The type of cutting tool, the cutting parameters, i.e., the feed rate, the cutting speed, and the cooling method and strategy [19] are very important when drilling takes place in plastics [20].

From a review of the literature, it is clear that conventional machining is still the major manufacturing process for many types of materials. The most popular machining operations are turning, milling [21,22,23], drilling [24], grinding, and polishing [25].

One of the problems discussed recently is drilling in components fabricated by additive manufacturing (AM). As drilling in 3D printed materials is becoming more and more common and it differs from drilling of traditional materials, it is important to analyze how the process parameters affect the properties of 3D prints. Research in this area is essential to improve the dimensional and geometric accuracy of the holes and their surface quality [26].

Another issue to be dealt with these days is drilling with tools made by additive manufacturing. This article describes drilling in polyamide using a drill bit fabricated by DMLM. Obviously, extensive research is required before 3D printed tools can be used for industrial applications. Investigations in this area have so far focused on milling or turning; the problems studied include cutting forces [27], surface integrity [28], tool wear [29], chip formation [30], surface roughness [31,32,33], vibration [34,35], and cooling methods [28,30]. Similar studies are necessary for additively manufactured tools used in drilling processes.

The problems discussed in this article have not been studied before. Machining with additively manufactured tools is a new area of research requiring a new approach to the operations and materials used in machining. The research results will contribute to the knowledge on both additive manufacturing and machining; they will provide some guidelines on the fabrication and use of 3D printed tools in different industrial applications.

The key objective of the study described in this article was to assess the potential use of a solid cutting tool (drill bit) 3D printed from 1.2709 steel using direct metal laser melting (DMLM), by comparing its performance with that of a commercially available tool manufactured in a conventional way. The study also aimed to compare the dimensional and geometric accuracy of the holes made with the two types of drill bits, as well as the forces and torques observed during drilling.

## 2. Materials and Methods

The experiments were carried out for two drill bits: one made of maraging steel 1.2709 3D printed through DMLM and the other, conventionally made of high-speed steel.

The 3D model of the additively manufactured twist drill bit was designed using Siemens NX. A Concept Laser M2 cusing DMLM-based machine was used for the printing. The process was performed in an argon atmosphere with an oxygen content of less than 0.1% for a layer thickness of 20 µm. The manufacturing time for one printed drill was approximately 20 h, and the cost was EUR 415. Maraging steel 1.2709 was used for the tool because of its excellent properties, for e.g., its high wear resistance, yield strength, and ultimate tensile strength, over a relatively wide range of temperatures. The drilling process generates friction and, consequently, high temperatures in the cutting zone. The chemical composition of the steel used for the tool is shown in Table 1.

**Figure 1:**
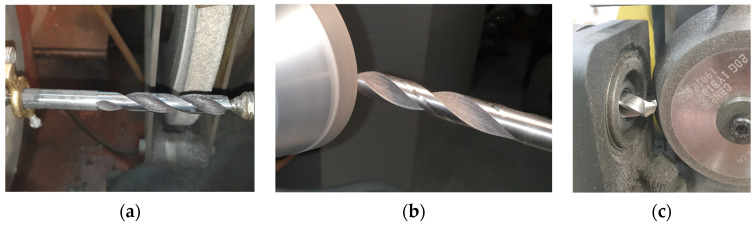
Equipment used for drill bit grinding: (**a**) Ponar-Tarnów RUP 28.45 FOS center grinding machine, (**b**) SACCKE grinding machine, (**c**) MRCM MR-20G drill bit sharpener.

As the 3D printed drill bit had a low dimensional and geometric accuracy and high surface roughness, the tool was ground using three different machines. The equipment used for this purpose is illustrated in Figure 1.

First, the shank and the face were ground using a Ponar-Tarnów RUP 28.45 FOS center grinding machine (Figure 1a). Then, the margin, the flank, and the face were shaped with a SAACKE UW-IC grinding machine (Figure 1b). Finally, the face and the chisel edge were corrected using an MRCM MR-20G drill bit sharpener (Figure 1c).

**Figure 2:**
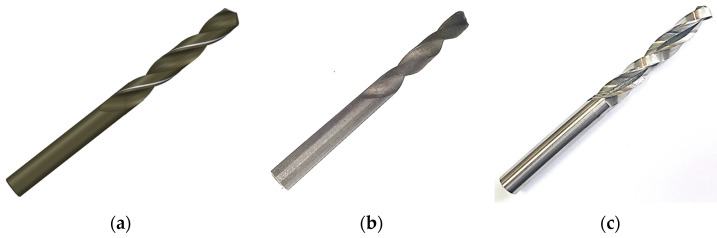
Stages of the 3D printing of the twist drill bit: (**a**) 3D model, (**b**) drill bit before grinding, (**c**) drill bit after grinding.

Figure 2 depicts the different stages in the fabrication of the twist drill bit. Figure 2a shows the 3D model used to print the steel 1.2709 drill bit by using DMLM technology (Figure 2b). Figure 2c shows the drill bit finished by grinding.

**Figure 3:**
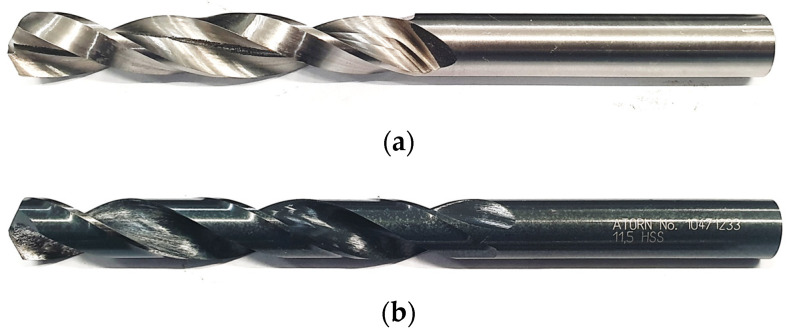
Types of drill bits: (**a**) 3D printed drill bit, (**b**) HK 11020480 HSS drill bit.

The dimensional and geometric accuracy of the 3D printed drill bit was assessed before and after the grinding processes. The equipment used for this purpose was a digital caliper (to measure the length of the printed tool before and after the finishing operations), Mahr Federal MarCal 16 EWV digital diameter measuring tool (to determine the drill bit diameter), a ZEISS O-INSPECT multisensory optical measuring machine (to measure the point angle), and a Taylor Hobson Talyrond 365 roundness measuring instrument (to measure the shank cylindricity error). The influence of the process parameters on the dimensional and geometric accuracy of the finished 3D printed drill bit (Figure 3a) was determined by analyzing the geometry and surface quality of the drill bit.

The other tool used for hole drilling was an HK 11020480 HSS drill bit by Atorn (Figure 3b). The tool has a point angle of 118°, a flute length of 94 mm, an overall length of 142 mm, and a cutting diameter of 11.5 mm.

The hole drilling tests were performed using 24 cylindrical specimens, 40 mm in diameter and 30 mm in height, made of a crystalline/semi-crystalline polymer called polyamide (PA), which is produced by polycondensation of hexamethylene diamine and adipic acid. The material has a density of approx. 1.14 g/cm³ and a melting point of about 255 °C. Polyamides are characterized by good mechanical properties, i.e., high tensile strength, good resistance to abrasion, and a low coefficient of friction. Another important benefit of PAs is their large dimensional stability at elevated temperatures. Because of their properties, PAs are used in a variety of industrial applications. They are commonly used in automotive and machine components, such as toothed wheels, bearings, bolts and nuts, pump components, couplings, cams, distributors, and drive shafts.

**Figure 4:**
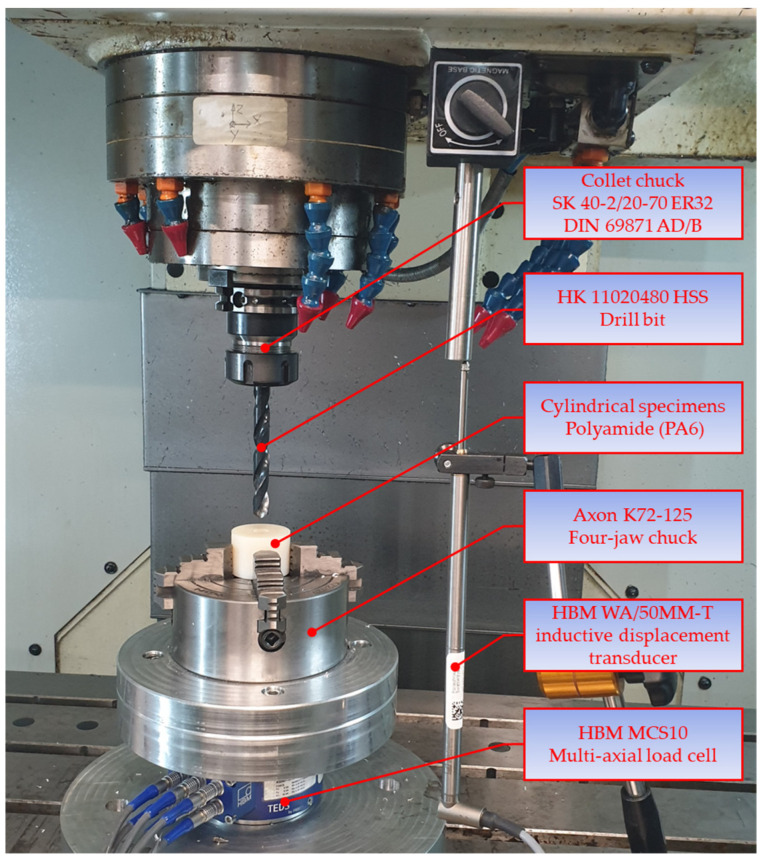
System for measuring the forces and torques during drilling.

The material, characterized by good mechanical stability, stiffness, damping capacity, and resistance to abrasion, is frequently used for machine elements because it is easy to machine. Each polyamide specimen was placed in an Axon K72-125 four-jaw chuck, mounted on the table of an FOP AVIA VMC 800 vertical milling center (Figure 4). The tests involved measuring the forces and torques by means of an HBM MCS10 multi-axial load cell. The force transducer was capable of simultaneously measuring the forces and torques along three axes (*x*, *y*, *z*).

The load cell provides real three-dimensional images of the forces and torques occurring during drilling. The MCS10 transducer was connected to each of the eight channels available on an HBM MX840B universal measuring amplifier equipped with a 24-bit analog-to-digital converter. The measuring system also included a HBM WA/50MM-T inductive displacement transducer, to measure the axial displacement of the tool during drilling to correlate the tool position with the instantaneous values of the forces and torques during drilling. The measurement process was coordinated by Catman data acquisition software.

The drilling tests using a VMC 800 vertical milling center did not involve applying any cutting fluid, which means that holes were drilled under dry lubrication conditions. Due to the prototypical nature of the printed drill bit, with no specific operating settings, the cutting speed and the feed rate were selected by following the recommendations of the producer of the HSS drill bit. The drilling was performed at two cutting speeds (20 and 30 m/min) and two feed rates (0.1 and 0.2 mm/rev). Each hole drilling test was run three times (Table 2).

All the signals providing information on the forces, torques, and tool displacements were filtered and analyzed using MATLAB.

As the signals representing the forces and torques during drilling were distorted signals, they were analyzed in the time and frequency domains. A fast Fourier transform (FFT) was used to convert the time-domain signals into frequency-domain signals. The Fourier transform X(jω) required to obtain the amplitude spectrum of the signal x(t) is given by the integral equation:

(1) $$X(j\omega )=\int_{-\infty }^{\infty }x(t)e^{-j\omega t}dt$$

where $j^{2}=-1$ is the imaginary unit and $e^{j\omega }=\cos\omega +j\sin\omega$.

For digitally processed signals, a numerical method known as a discrete Fourier transform (DFT) is used. An efficient algorithm for the fast Fourier transform (FFT) is used to calculate the DFT. The DFT can be expressed as:

(2) $$X(k)=\sum_{n=0}^{N-1}x(n)e^{-j2\pi kn/N},\;n=0,\ldots N-1,\;k=0,\ldots N-1$$

where N is the number of specimens.

The discrete value X(k) represents a complex number that provides information about the signal amplitude and phase. Under industrial conditions, the amplitude spectrum is usually determined as:

(3) $$A(k)=\left\{\begin{array}{l} \frac{1}{N}\left|X(0)\right|,\;k=0\\ \frac{2}{N}\left|X(k)\right|,\;k=1,\ldots ,N/2 \end{array}\right.$$

The values of A(k) and x(n) have the same physical dimension.

**Figure 5:**
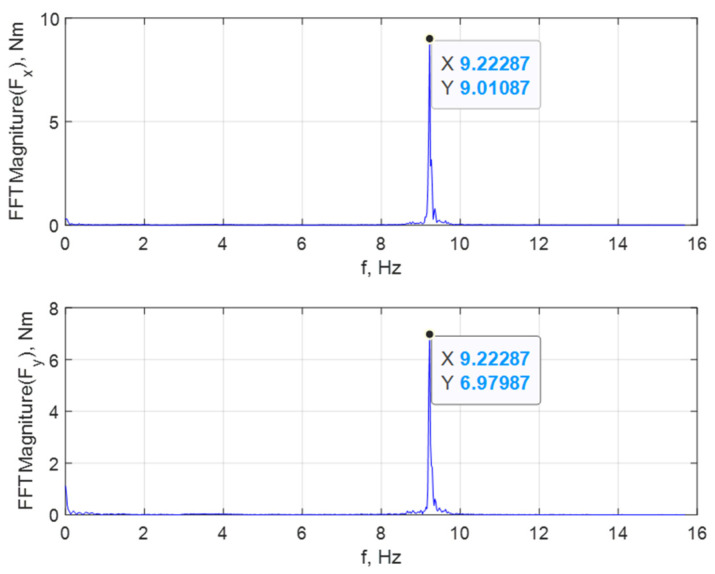
Amplitude spectra of the forces in the *x* and *y* directions.

This article analyzes the amplitude spectra to determine the forces and torques occurring during drilling. For each measured signal, an FFT algorithm with a sampling frequency of 19.2 kHz was used to obtain a useful range of frequency from 0 to 9.6 kHz (according to the Nyquist–Shannon sampling theorem, also known as the Kotielnikow–Shannon theorem). For the analyzed force and torque signals, the proper frequency peaks were found for the drill bit revolution frequency. Figure 5 shows the forces, respectively, in the *x*, *y* and *z* directions and the amplitude spectra of the forces in the *x* and *y* directions, calculated on the basis of the FFT analysis for the drilling performed at a cutting speed of 548 (554) rev/min.

**Figure 6:**
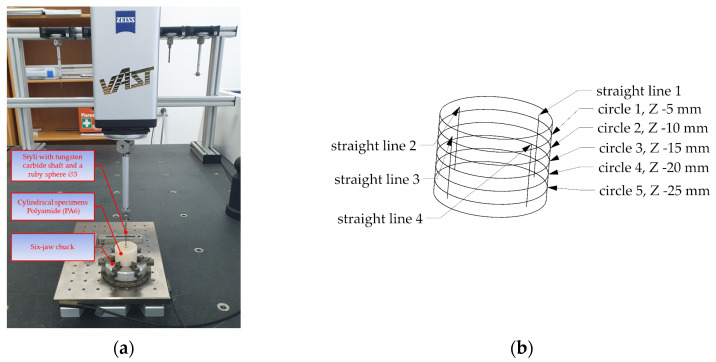
Measurement of the geometric and dimensional accuracy of holes: (**a**) measuring system, (**b**) measurement strategy.

The dimensional and geometric accuracy of the holes was assessed using a ZEISS Prismo Navigator coordinate measuring machine (Figure 6a). Figure 6b shows the measurement strategy to assess the hole accuracy. The straightness error was determined by measuring the generating lines in four angular positions, spaced every 90° using a Gaussian filter with λc = 2.5 mm. The roundness error was calculated on the basis of 1500 points measured in five cross-sections, i.e., at depths of 5 mm, 10 mm, 15 mm, 20 mm, and 25 mm, using a Gaussian 2-15 UPR filter.

The measurements were performed using a ZEISS VAST scanning probe with a ruby ball 3 mm in diameter, at a scanning speed of 5 mm/s.

## 3. Results and Discussion

The influence of the manufacturing process on the dimensional and geometric accuracy of the finished 3D printed drill bit was determined by measuring its key parameters. The tool after grinding had a point angle of 118°, a flute length of 71 mm, an overall length of 134.5 mm, and a cutting diameter of 11.5 mm. The drill bit weighed 77.9 g.

The conclusions concerning the differences between the 3D model and the finished drill bit were used to prepare guidelines on the fabrication and use of tools 3D printed from metal powders. Table 3 compares the basic parameters of the 3D model with those of the printed tool after grinding.

From the measurement data in Table 3, it is evident that at the design stage the CAD model of the drill bit needs to include material allowances because of the inaccuracy of the printing process. The minimum radius allowance for a drill bit with a diameter of 12 mm should be 0.25 mm per side, with the total being 0.5 mm. This value seems sufficient to achieve high geometric and dimensional accuracy of the shank, the face, and the flank. Unfortunately, with this radius allowance it is not possible to grind the whole surface of the flute; some areas remain unfinished, as illustrated in Figure 2c. For this reason, it is recommended that at the design stage the radius allowance in the flute area should be increased to a minimum of 0.5 mm per side. While selecting the length allowances (along the tool axis), a designer should add 0.5−1 mm for facing and approximately 5 mm per center hole for center grinding. If the finishing process involves center grinding, it is not recommended to design a drill bit with a tip because it will have to be removed to make a center hole. In this study, the difference in the overall length between the printed tool and the commercially available tool was 15 mm. The length allowances were necessary for the following operations: first to create the center holes at both ends (2.5 mm × 2), so that the center grinding could be performed, and then to shape the drill bit tip (10 mm).

The dimensional and geometric accuracy of the shank of the 3D printed tool was measured before and after grinding. The measurements were taken in five cross-sections along the shank using a Talyrond 365. The results are shown in Figure 6 and Table 4.

**Figure 7:**
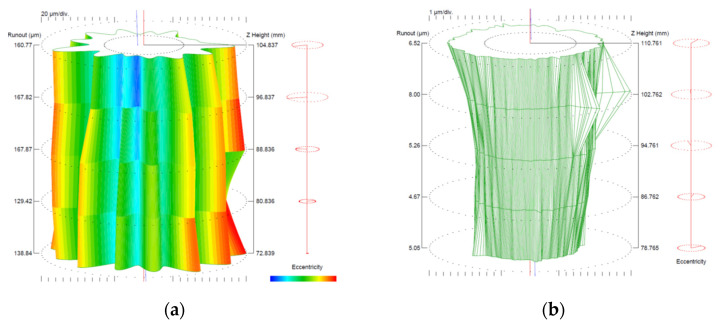
Cylindricity of the 3D printed shank: (**a**) before and (**b**) after grinding.

From the measurement data obtained for the shank of the 3D printed tool, displayed in Figure 7 and Table 4, it is clear that the cylindricity error was 171.4 μm. The cylindricity error was mainly due to the form distortion (twelve lobes) observed in the cross-sections.

The finishing by center grinding reduced the cylindricity error of the tool shank to 7.6 μm. The cylindricity error of the shank after grinding resulted from the conicity error. The analysis of the shank cylindricity error confirmed the assumption that the minimum radius allowance should be 0.25 mm per side.

The next step was to assess the dimensional and geometric accuracy of holes made with the two types of drill bits. Table 5 shows the mean values of the errors obtained with a ZEISS Prismo Navigator coordinate measuring machine. Each finished specimen was measured in five cross-sections and, then, the mean cylindricity error of the hole was determined.

Another important parameter describing the drilling accuracy is the hole straightness. It was determined in four angular positions spaced every 90°. The measurement strategy is illustrated in Figure 6.

The values provided in Table 5 indicate that for holes drilled at v_c_ = 30 m/min and f_n_ = 0.2 mm/rev, all errors, apart from roundness 1, were smaller for the additively manufactured tool when compared with the commercially made tool. It can, thus, be concluded that the holes drilled with the 3D printed drill bit were characterized by lower roundness, cylindricity, and straightness errors. 

The lowest roundness error of 0.019 mm was obtained for holes drilled with the 3D printed tool at v_c_ = 20 m/min and f_n_ = 0.2 mm/rev, and at v_c_ = 30 m/min and f_n_ = 0.1 mm/rev. For the conventionally made drill bit, the lowest roundness error of 0.037 mm was observed at v_c_ = 30 m/min and f_n_ = 0.2 mm/rev; the error was 0.018 mm larger than that reported for the printed tool. 

The hole cylindricity data suggest that the lowest error of 0.064 mm was reported for the 3D printed drill bit operating at v_c_ = 20 m/min and f_n_ = 0.2 mm/rev. For the HSS drill bit, the lowest cylindricity error was 0.031 mm larger; it reached 0.095 mm at v_c_ = 30 m/min and f_n_ = 0.2 mm/rev.

The lowest hole straightness error from drilling with the 3D printed tool was 0.021 mm and it was obtained at v_c_ = 20 m/min and f_n_ = 0.2 mm/rev. For the HK 11020480 HSS drill bit, the lowest hole straightness error was 0.021 mm larger, reaching 0.042 mm.

Table 6 shows the mean square values of the forces at the *x, y* and *z* axes and the torque at the *z* axis measured during the hole drilling tests.

The results reveal that the mean values of the forces at the *x* and *y* axes and the torque at the *z* axis (F_x_—radial force, F_y_—tangential force, F_z_—axial force) were lower for the 3D printed tool when compared with those obtained for the HSS tool. However, the mean values of the force acting at the *z* axis were larger for the printed drill bit than for the HSS drill bit. The lowest mean value of the force at the *x* axis reported for the printed drill bit was 7.16 N at v_c_ = 20 m/min and f_n_ = 0.2 mm/rev. The highest mean values of the forces acting at the *x* and *y* axes at v_c_ = 30 m/min and f_n_ = 0.2 mm/rev were 10.96 N and 10.22 N, respectively. The lowest mean value of the force at the *y* axis was 7.55 N at v_c_ = 20 m/min and f_n_ = 0.1 mm/rev. The lowest mean values of the forces at the *x* and *y* axes reported for the HSS drill bit operating at v_c_ = 20 m/min and f_n_ = 0.1 mm/rev were 11.06 N and 11.07 N, respectively. The lowest mean value of the torque reported for the printed drill bit was 0.40 Nm at v_c_ = 20 m/min and f_n_ = 0.1, 0.2 mm/rev. The highest mean values of the torque acting at v_c_ = 30 m/min and f_n_ = 0.2 mm/rev was 1.75 Nm. The lowest mean values of the torque for the HSS drill bit operating at v_c_ = 20 m/min and f_n_ = 0.1 mm/rev was 0.49 Nm, and the highest mean values acting at v_c_ = 30 m/min and f_n_ = 0.1, 0.2 mm/rev was 1.75 Nm.

**Figure 8:**
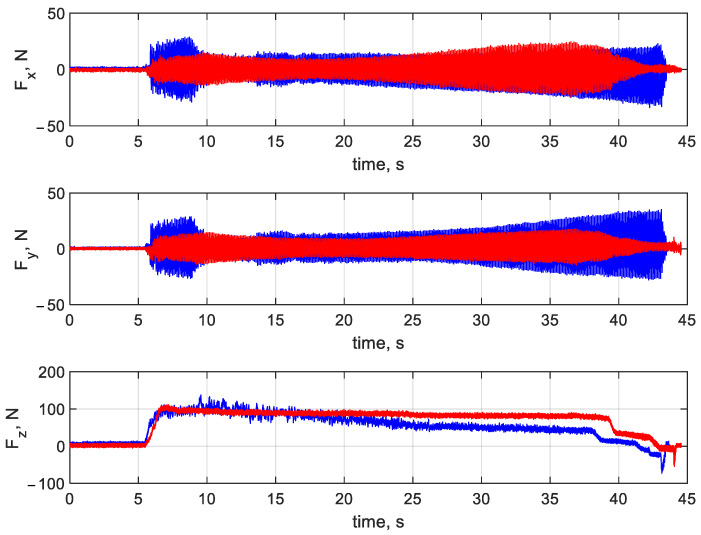
Forces at the *x, y,* and *z* axes at v_c_ = 20 m/min and f_n_ = 0.1 mm/rev (the printed tool in red and the HSS tool in blue).

Figure 7 and Figure 8 illustrate the forces and torques, respectively, at the *x, y,* and *z* axes for drilling at v_c_ = 20 m/min and f_n_ = 0.1 mm/rev. Red represents the printed drill bit, while blue the HSS drill bit. In the diagrams in Figure 7 and Figure 8, three characteristic stages can be distinguished. Stage 1 corresponds to the beginning of the drilling process (from second 6 until second 10), when the drill bit enters and gradually cuts into the workpiece until the full cutting diameter is reached. During stage 2 (from second 10 to second 39), the full diameter of the drill bit is engaged in the hole cutting. At stage 3 (from second 39 until the end), the drill bit exits the workpiece and returns to the starting position.

From Figure 8, it is clear that there are differences in performance between the printed tool and the HSS tool. For the conventionally made drill bit, forces acting on the system at the *x* and *y* axes are larger when the tool enters the workpiece; this stage lasts about 10 s longer than for the printed tool. Then, the forces stabilize and are similar for both tools. The diagrams in Figure 8 suggest smaller hole straightness and roundness errors for the printed tool. The forces acting at the *z* axis are more stable (with curves more linear in shape) for the printed drill bit rather than for the drill bit produced by Atron.

**Figure 9:**
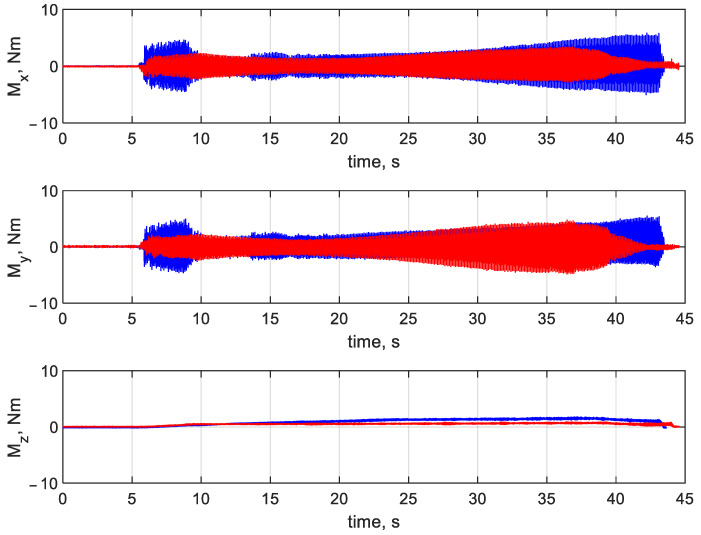
Torques at the *x, y,* and *z* axes at v_c_ = 20 m/min and f_n_ = 0.1 mm/rev (the printed tool in red and the HSS tool in blue).

Analysis of Figure 9 reveals that diagrams of the torques at the *x* and *y* axes for drilling at v_c_ = 20 m/min and f_n_ = 0.1 mm/rev are similar to the diagrams of the forces (Figure 7). The signals registered in drilling at v_c_ = 20 m/min and f_n_ = 0.1 mm/rev show that the torque at the *z* axis was larger for the conventionally made drill bit.

## 4. Conclusions

The main aim of this article was to compare the performance of two twist drill bits: a conventionally made HK 11020480 HSS drill bit and a specially designed 3D printed drill bit fabricated by DMLM using maraging steel 1.2709. The hole roundness, cylindricity, and straightness errors were also analyzed.

The results of the comparative study can be summarized as follows:

- Three-dimensional (3D) printed steel 1.2709 drill bits are suitable for drilling holes in polyamide 6 (PA6) and can be used as an alternative to HSS steel drill bits in the test range conducted;
- Material allowances are required for 3D printed drill bits because of the insufficient accuracy of the AM process. The allowances need to be taken into account at the design stage while creating a CAD model. The minimum radius allowance for a drill bit 12 mm in diameter is 0.25 mm per side to achieve high geometric and dimensional accuracy of the cutting tool;
- The length allowances, i.e., those along the tool axis, are required for center grinding (5 mm for a center hole at each end) and for facing (0.5−1 mm);
- The dimensional and geometric accuracy of the holes made in the polyamide 6 (PA6) by the specially designed 3D printed drill bit were higher than that reported for the HSS drill bit;
- The lowest hole roundness error of 0.019 mm was obtained for the 3D printed drill bit, when the conventionally made drill bit was used, the lowest hole roundness error was 0.018 mm higher;
- The lowest hole straightness error reported for the 3D printed drill bit was 0.021 mm and it was achieved at vc = 20 m/min and fn = 0.2 mm/rev. By contrast, the lowest hole straightness error obtained with the same process parameters for the HK 11020480 HSS drill bit was 0.042 mm, which was twice as high as that obtained for the 3D printed tool;
- The lowest hole cylindricity error measured for the 3D printed drill bit was 0.064 mm, for the HSS tool this parameter was 0.031 mm higher;
- For the 3D printed drill bit, the lowest and the highest mean values of the force acting at the x axis were 7.16 N and 10.96 N, respectively; for the force acting at the y axis, the values were 7.55 N and 10.22 N, respectively. The lowest mean square values of the forces at the *x* and *y* axes reported for the HSS drill bit were 11.06 N and 11.07 N, respectively, while the highest were 11.61 and 11.51 N, respectively.

## Figures and Tables

**Table 1:** Composition of steel 1.2709.

Chemical Composition of Steel 1.2709 in wt.%														
Workpiece Material	Aluminum, Al	Carbon, C	Cobalt, Co	Chromium, Cr	Manganese, Mn	Molybdenum, Mo	Nitrogen, N	Nickel, Ni	Oxygen, O	Phosphorus, P	Sulphur, S	Silicon, Si	Titanium, Ti	Iron, Fe
Max	0.1	0.03	10	0.25	0.15	5.2	0.1	19	0.1	0.01	0.01	0.1	1.2	≈65
Min	-	-	8.5	-	-	4.5	-	17	-	-	-	-	0.8	

**Table 2:** Test conditions.

Specimen	Cutting Speed v_c_, m/min	Feed per Revolution f_n_, mm/rev	Spindle Speed n, rev/min	Feed Rate v_f_, mm/min	Drill Bit Type
1,2,3	20	0.1	554	55.4	Printed
4,5,6	20	0.2	554	110.8	Printed
7,8,9	30	0.1	830	83	Printed
10,11,12	30	0.2	830	166	Printed
13,14,15	20	0.1	554	55.4	HK 11020480 HSS
16,17,18	20	0.2	554	110.8	HK 11020480 HSS
19,20,21	30	0.1	830	83	HK 11020480 HSS
22,23,24	30	0.2	830	166	HK 11020480 HSS

**Table 3:** Parameters of the CAD model and the finished tool.

Parameter	CAD Model	3D Printed Tool after Grinding	Difference
Drill bit diameter, mm	12	11.5	0.5
Overall length, mm	149.5	134.5	15
Flute length, mm	79.5	71	8.5
Point angle, °	118	118	0

**Table 4:** Measurement data concerning the cylindricity of the drill bit shank.

Parameter	Printed Tool before Grinding	Printed Tool after Grinding
CYLp, µm	75.37	5.52
CYLv, µm	96.04	2.13
CYLt, µm	171.43	7.65

Where: CYLp—cylinder peak—the value of the largest positive local cylindrical deviation from the least squares reference cylinder, CYLv—cylinder valley—the absolute value of the largest negative local cylindrical deviation from the least squares reference cylinder, CYLt—cylinder peak to valley—the radial separation of two cylinders, coaxial with the fitted reference axis, which totally enclose the measured data.

**Table 5:** Average values of the geometric errors of the holes, obtained by the drill bits.

Error		3D Printed Drill Bit				HK 11020480 HSS Drill Bit			
		v_c_ = 20 m/min, f_n_ = 0.1 mm/rev	v_c_ = 20 m/min, f_n_ = 0.2 mm/rev	v_c_ = 30 m/min, f_n_ = 0.1 mm/rev	v_c_ = 30 m/min, f_n_ = 0.2 mm/rev	v_c_ = 20 m/min, f_n_ = 0.1 mm/rev	v_c_ = 20 m/min, f_n_ = 0.2 mm/rev	v_c_ = 30 m/min, f_n_ = 0.1 mm/rev	v_c_ = 30 m/min, f_n_ = 0.2 mm/rev
Roundness 1	mm	0.042	0.031	0.061	0.048	0.165	0.042	0.074	0.046
Roundness 2	mm	0.040	0.025	0.047	0.039	0.072	0.049	0.101	0.048
Roundness 3	mm	0.029	0.021	0.037	0.032	0.065	0.041	0.075	0.042
Roundness 4	mm	0.026	0.019	0.019	0.027	0.055	0.038	0.054	0.037
Roundness 5	mm	0.023	0.022	0.019	0.022	0.046	0.057	0.078	0.037
Cylindricity	mm	0.086	0.064	0.110	0.084	0.169	0.119	0.161	0.095
Straightness 1	mm	0.025	0.021	0.046	0.027	0.076	0.054	0.084	0.050
Straightness 2	mm	0.025	0.023	0.044	0.029	0.074	0.051	0.086	0.042
Straightness 3	mm	0.029	0.022	0.043	0.028	0.071	0.053	0.079	0.054
Straightness 4	mm	0.024	0.022	0.038	0.028	0.085	0.056	0.069	0.047

**Table 6:** Mean square values of cutting the force and torque obtained for the two drill bits.

			F_x_	F_y_	F_z_	M_z_
			N	N	N	Nm
3D printed drill bit	v_c_ = 20 m/min, f_n_ = 0.1 mm/rev	1	8.23	6.93	75.43	0.41
		2	7.83	7.05	74.89	0.40
		3	7.40	8.66	73.96	0.39
		mean	7.82	7.55	74.76	0.40
	v_c_ = 20 m/min, f_n_ = 0.2 mm/rev	1	7.40	8.66	73.96	0.39
		2	7.05	9.32	124.41	0.41
		3	7.03	8.68	121.81	0.41
		mean	7.16	8.89	106.73	0.40
	v_c_ = 30 m/min, f_n_ = 0.1 mm/rev	1	8.91	10.16	66.93	0.85
		2	11.71	10.04	61.35	0.85
		3	12.25	10.46	67.52	0.85
		mean	10.96	10.22	65.27	0.85
	v_c_ = 30 m/min, f_n_ = 0.2 mm/rev	1	6.17	6.90	75.74	1.64
		2	7.35	11.50	116.50	1.29
		3	12.11	9.42	116.48	2.33
		mean	8.54	9.27	102.91	1.75
HK 11020480 HSS drill bit	v_c_ = 20 m/min, f_n_ = 0.1 mm/rev	1	11.29	11.56	63.29	0.52
		2	11.14	10.54	59.12	0.50
		3	10.74	11.09	55.88	0.48
		mean	11,06	11,07	59.43	0.50
	v_c_ = 20 m/min, f_n_ = 0.2 mm/rev	1	10.77	11.60	76.06	0.49
		2	11.42	11.35	73.43	0.49
		3	11.08	11.60	73.16	0.48
		mean	11,09	11.51	74.22	0.49
	v_c_ = 30 m/min, f_n_ = 0.1 mm/rev	1	11.58	10.71	57.55	0.88
		2	10.68	11.52	57.99	0.87
		3	11.06	11.33	57.30	0.87
		mean	11.11	11.19	57.61	0.87
	v_c_ = 30 m/min, f_n_ = 0.2 mm/rev	1	11.39	11.98	72.14	0.88
		2	11.77	11.10	73.41	0.87
		3	11.67	11.46	72.97	0.87
		mean	11.61	11.51	72.84	0.87

## Data Availability

Data is contained within the article.

## References

[1] Scholz S.G., Howlett R.J., Setchi R. (2022). Sustainable Design and Manufacturing.

[2] Charles A., Hofer A., Elkaseer A., Scholz S.G., Scholz S.G., Howlett R.J., Setchi R. (2022). Additive Manufacturing in the Automotive Industry and the Potential for Driving the Green and Electric Transition. Sustainable Design and Manufacturing.

[3] Zhang X., Liang E. (2019). Metal additive manufacturing in aircraft: Current application, opportunities and challenges. IOP Conf. Ser. Mater. Sci. Eng..

[4] Vandenbroucke B., Kruth J.-P. (2007). Selective laser melting of biocompatible metals for rapid manufacturing of medical parts. Rapid Prototyp. J..

[5] Liu J., Sun Q., Zhou C., Wang X., Li H., Guo K., Sun J. (2019). Achieving Ti6Al4V alloys with both high strength and ductility via selective laser melting. Mater. Sci. Eng. A.

[6] Dimov S.S., Pham D.T., Lacan F., Dotchev K.D. (2001). Rapid tooling applications of the selective laser sintering process. Assem. Autom..

[7] Tay F., Haider E.A., Rahman M., Lee J.Y., Ong T. (2000). Manufacture of RP tools with high quality surface finish using high temperature epoxy resin and electroless nickel plating. Surf. Eng..

[8] Mathew N.T., Vijayaraghavan L. (2017). High-throughput dry drilling of titanium aluminide. Mater. Manuf. Process..

[9] Rysava Z., Bruschi S., Carmignato S., Medeossi F., Savio E., Zanini F. (2016). Micro-drilling and Threading of the Ti6Al4V Titanium Alloy Produced through Additive Manufacturing. Procedia CIRP.

[10] Karabulut Y., Kaynak Y. (2020). Drilling process and resulting surface properties of Inconel 718 alloy fabricated by Selective Laser Melting Additive Manufacturing. Procedia CIRP.

[11] Habib N., Sharif A., Hussain A., Aamir M., Giasin K., Pimenov D.Y., Ali U. (2021). Analysis of Hole Quality and Chips Formation in the Dry Drilling Process of Al7075-T6. Metals.

[12] Waqar S., Asad S., Ahmad S., Abbas C.A., Elahi H. (2016). Effect of Drilling Parameters on Hole Quality of Ti-6Al-4V Titanium Alloy in Dry Drilling. Mater. Sci. Forum.

[13] Ming W., Dang J., An Q., Chen M. (2020). Chip formation and hole quality in dry drilling additive manufactured Ti6Al4V. Mater. Manuf. Process..

[14] Balaji M., Venkata Rao K., Mohan Rao N., Murthy B. (2018). Optimization of drilling parameters for drilling of TI-6Al-4V based on surface roughness, flank wear and drill vibration. Measurement.

[15] Dang J., Liu G., Chen Y., An Q., Ming W., Chen M. (2019). Experimental investigation on machinability of DMLS Ti6Al4V under dry drilling process. Mater. Manuf. Process..

[16] Kivak T., Şeker U. (2015). Effect of cryogenic treatment applied to M42 HSS drills on the machinability of Ti-6Al-4V alloy. Mater. Tehnol..

[17] Nam J., Lee S.W. (2018). Machinability of titanium alloy (Ti-6Al-4V) in environmentally-friendly micro-drilling process with nanofluid minimum quantity lubrication using nanodiamond particles. Int. J. Precis. Eng. Manuf.-Green Technol..

[18] Zhu Z., Guo K., Sun J., Li J., Liu Y., Chen L., Zheng Y. (2018). Evolution of 3D chip morphology and phase transformation in dry drilling Ti6Al4V alloys. J. Manuf. Process..

[19] Pimenov D.Y., Mia M., Gupta M.K., Machado A.R., Tomaz Í.V., Sarikaya M., Wojciechowski S., Mikolajczyk T., Kapłonek W. (2021). Improvement of machinability of Ti and its alloys using cooling-lubrication techniques: A review and future prospect. J. Mater. Res. Technol..

[20] Quadrini F. (2008). Machining of plastics: A new approach for modeling. Polym. Eng. Sci..

[21] Nowakowski L., Skrzyniarz M., Miko E., Takosoglu J., Blasiak S., Laski P., Bracha G., Pietrala D., Zwierzchowski J., Blasiak M., Dancova P. (2017). Influence of the cutting parameters on the workpiece temperature during face milling. Experimental Fluid Mechanics 2016 (EFM16).

[22] Nowakowski L., Bartoszuk M., Skrzyniarz M., Blasiak S., Vasileva D. (2022). Influence of the Milling Conditions of Aluminium Alloy 2017A on the Surface Roughness. Materials.

[23] Nowakowski L., Skrzyniarz M., Blasiak S., Bartoszuk M. (2020). Influence of the Cutting Strategy on the Temperature and Surface Flatness of the Workpiece in Face Milling. Materials.

[24] Uysal A. (2015). Relation between Drill Bit Temperature and Chip Forms in Drilling of Carbon Black Reinforced Polyamide. J. Therm. Eng..

[25] Sari N.M.G.A.W., Fardaniah S., Masulili C. (2017). Color changing in denture base polyamide 12 and polyamide microcrystalline after polishing in laboratory and dental clinic. J. Phys. Conf. Ser..

[26] Campos Rubio J.C., Da Silva L.J., Leite W.d.O., Panzera T.H., Filho S.L.M.R., Davim J.P. (2013). Investigations on the drilling process of unreinforced and reinforced polyamides using Taguchi method. Compos. Part B Eng..

[27] Bonaiti G., Parenti P., Annoni M., Kapoor S. (2017). Micro-milling Machinability of DED Additive Titanium Ti-6Al-4V. Procedia Manuf..

[28] Bordin A., Medeossi F., Ghiotti A., Bruschi S., Savio E., Facchini L., Bucciotti F. (2016). Feasibility of Cryogenic Cooling in Finishing Turning of Acetabular Cups Made of Additive Manufactured Ti6Al4V. Procedia CIRP.

[29] Sartori S., Moro L., Ghiotti A., Bruschi S. (2017). On the tool wear mechanisms in dry and cryogenic turning Additive Manufactured titanium alloys. Tribol. Int..

[30] Bordin A., Sartori S., Bruschi S., Ghiotti A. (2017). Experimental investigation on the feasibility of dry and cryogenic machining as sustainable strategies when turning Ti6Al4V produced by Additive Manufacturing. J. Clean. Prod..

[31] Gong Y., Li P. (2019). Analysis of tool wear performance and surface quality in post milling of additive manufactured 316L stainless steel. J. Mech. Sci. Technol..

[32] Li F., Chen S., Shi J., Tian H., Zhao Y. (2017). Evaluation and Optimization of a Hybrid Manufacturing Process Combining Wire Arc Additive Manufacturing with Milling for the Fabrication of Stiffened Panels. Appl. Sci..

[33] Zhang S., Zhang Y., Gao M., Wang F., Li Q., Zeng X. (2019). Effects of milling thickness on wire deposition accuracy of hybrid additive/subtractive manufacturing. Sci. Technol. Weld. Join..

[34] Skrzyniarz M., Nowakowski L., Miko E., Borkowski K. (2021). Influence of Relative Displacement on Surface Roughness in Longitudinal Turning of X37CrMoV5-1 Steel. Materials.

[35] Shi H., Song F., Fu L. (2011). Experimental study on drilling force in printed circuit board micro drilling process. Circuit World.

